# The relationship between COVID‐19 viral load and disease severity: A systematic review

**DOI:** 10.1002/iid3.580

**Published:** 2021-12-13

**Authors:** Omid Dadras, Amir M. Afsahi, Zahra Pashaei, Hengameh Mojdeganlou, Amirali Karimi, Pedram Habibi, Alireza Barzegary, Amirata Fakhfouri, Pegah Mirzapour, Nazanin Janfaza, Soheil Dehghani, Fatemeh Afroughi, Mohsen Dashti, Sepideh Khodaei, Esmaeil Mehraeen, Fabricio Voltarelli, Jean‐Marc Sabatier, SeyedAhmad SeyedAlinaghi

**Affiliations:** ^1^ The Excellent Center for Dengue and Community Public Health (EC for DACH), School of Public Health Walailak University Nakhon Si Thammarat Thailand; ^2^ Department of Radiology, School of Medicine University of California, San Diego (UCSD) La Jolla California USA; ^3^ Iranian Research Center for HIV/AIDS, Iranian Institute for Reduction of High‐Risk Behaviors Tehran University of Medical Sciences Tehran Iran; ^4^ Department of Pathology Urmia University of Medical Sciences Urmia Iran; ^5^ School of Medicine Tehran University of Medical Sciences Tehran Iran; ^6^ School of Medicine Islamic Azad University Tehran Iran; ^7^ Internal Medicine Department, Imam Khomeini Hospital Complex, School of Medicine Tehran University of Medical Sciences Tehran Iran; ^8^ Pars Hospital Iran University of Medical Sciences Tehran Iran; ^9^ Department of Radiology Tabriz University of Medical Sciences Tabriz Iran; ^10^ Department of Health Information Technology Khalkhal University of Medical Sciences Khalkhal Iran; ^11^ Graduation Program of Health Sciences, Faculty of Medicine Federal University of Mato Grosso Cuiabá Brazil; ^12^ Université Aix‐Marseille Institut deNeuro‐physiopathologie (INP) UMR 7051, Faculté de Pharmacie Marseille France

**Keywords:** COVID‐19, prognosis, SARS‐CoV‐2, severity, viral load

## Abstract

**Introduction:**

Patients with COVID‐19 may present different viral loads levels. However, the relationship between viral load and disease severity in COVID‐19 is still unknown. Therefore, this study aimed to systematically review the association between SARS‐CoV‐2 viral load and COVID‐19 severity.

**Methods:**

The relevant studies using the keywords of “COVID‐19” and “viral load” were searched in the databases of PubMed, Scopus, Google Scholar, and Web of Science. A two‐step title/abstract screening process was carried out and the eligible studies were included in the study.

**Results:**

Thirty‐four studies were included from the initial 1015 records. The vast majority of studies have utilized real‐time reverse transcription‐polymerase chain reaction of the nasopharyngeal/respiratory swabs to report viral load. Viral loads were commonly reported either as cycle threshold (*C*_t_) or log_10_ RNA copies/ml.

**Conclusion:**

The results were inconclusive about the relationship between COVID‐19 severity and viral load, as a similar number of studies either approved or opposed this hypothesis. However, the studies denote the direct relationship between older age and higher SARS‐CoV‐2 viral load, which is a known risk factor for COVID‐19 mortality. The higher viral load in older patients may serve as a mechanism for any possible relationships between COVID‐19 viral load and disease severity. There was a positive correlation between SARS‐CoV‐2 viral load and its transmissibility. Nonetheless, further studies are recommended to precisely characterize this matter.

## Graphical abstract

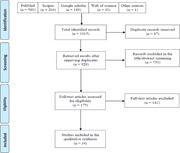

## 1 INTRODUCTION

Coronavirus disease 2019 (COVID‐19) is an acute respiratory syndrome caused by coronavirus 2 (SARS‐CoV‐2) that causes inflammation and multiorgan involvement in the body.^1^, ^2^, ^3^ The World Health Organization (WHO) declared this disease as a “public health emergency of international concern” on January 30, 2020.^4^, ^5^ As of September 3, 2021, there have been more than 218 million confirmed cases of COVID‐19 and 4,526,583 death have been reported around the world.^6^, ^7^

When SARS‐CoV‐2 enters lung cells, it attacks the lower respiratory tract and attaches strongly to its receptors in the lungs; namely, angiotensin‐converting enzyme receptors.^8^, ^9^ When an infection in the lower respiratory tract activates immune cells such as neutrophils and macrophages, it releases several chemokines and cytokines that activate the immune system like B and T cells, this irregular response eventually leads to elevated levels of cytokines, called cytokine storms or hypercytokinemia.^10^ As a result, severe pneumonia involving various organs could develop that cause diverse symptoms and signs as well as consequent psychological harm.^1^ The symptoms of COVID‐19 are fever, dry cough, dyspnea, headache, fatigue, loss of taste and/or smell, and gastrointestinal symptoms.^11^ In laboratory results the liver enzymes are high, lymphocytes are low (lymphocytopenia), and C‐reactive protein levels are high. Eventually, the virus causes acute respiratory distress syndrome that may lead to death.^12^ SARS‐CoV‐2 belongs to the Nidovirales order, Coronaviridae family, Coronavirinae subfamily, it is an enveloped virus with a positive‐sense, single‐stranded RNA genome of approximately 30 kb.^13^

Since its emergence, the SARS‐CoV‐2 has undergone multiple mutations resulting in weaker or even or more dangerous variants of the virus. SARS‐CoV‐2 continuously evolves and potentially becomes more transmissible or fatal with each mutation.^2^ Four variants of SARS‐CoV‐2 have been declared as the “variants of concern” by the WHO so far, which cause COVID‐19.

A. Alpha variant: Alpha variant, or the lineage B.1.1.7, is the first SARS‐CoV‐2 variant and can be substituted by 23 mutations. As a consequence of the mutation, the transmissibility of the virus increased by about 50% as compared to the wild strain, making it more infectious with more severe complications^14^;

B. Beta variant: These mutations enhance the ability of the virus to attach to the human cells more easily in comparison with the previous variants^15^;

C. Gamma variant: Gamma variant caused widespread infection in early 2021 and is currently considered as a “variant of concern”^16^;

D. Delta variant: The Delta variant is more infectious and each infected person can transmit the virus to seven or more people.^17^

For the clinical management of COVID‐19 disease, it is substantial to quantify the viral load of the blood.^18^ Viral load indicates active viral proliferation and is used to identify the severe viral infections of the respiratory tract and monitor the disease progression and treatment.^19^ The viral load can be obtained from the patient's viral RNA with a certain concentration (the value that exceeds the threshold) by testing the value of the *C*_t_ cycle threshold of the reverse transcription‐polymerase chain reaction (RT‐PCR). The lower the *C*_t_ values than a patient's sample, the higher the viral load.^20^ The relationship between the viral load and severity of disease in COVID‐19 patients has not yet been fully understood. The investigation demonstrated that patients with COVID‐19 who have been treated in the intensive care unit with a severe illness have a relatively higher viral load. A study also suggested that in large hospital groups, a high viral load is associated with an increased risk of death.^21^ Thus, the study of the correlation between COVID‐19 viral load and the progression of the disease and the treatment and prevention of COVID‐19 helps to science promotion significantly.^22^

A Chinese study working on the association of viral load with the development of COVID‐19 found that patients with more viral load had fewer lymphocytes but more neutrophils.^23^ In another study that examined the relationship between viral load and disease severity with COVID‐19 clinical results, viral load in severe disease was much higher than in mild or asymptomatic disease.^24^ However, conflicts exist regarding the effects of SARS‐CoV‐2 viral load on disease severity. Therefore, the present study systematically reviewed the association between SARS‐CoV‐2 viral load and COVID‐19 severity.

## 2 METHODS

### 2.1 Data sources

Relevant articles were systematically searched from the keywords “COVID‐19” and “viral load” in the online databases of PubMed, Science Direct, Scopus, and Web of Science. All the relevant literature published from December 2019 to August 2021 was retrieved and further screened using EndNote.

### 2.2 Study objectives

The principal aim was to investigate the relationship between the COVID‐19 viral load and its severity. However, the relationship between viral load and COVID‐19 infectivity as well as the patients' age and viral load was also discussed.

### 2.3 Study selection and inclusion/exclusion criteria

We conducted a two‐phase screening process; first, the studies were evaluated based on their title and abstract, and then the eligible ones were screened based on their full texts. We included peer‐reviewed articles that studied the association between SARS‐CoV‐2 viral load and the COVID‐19 disease severity or mortality. The selected articles were cross‐examined by other researchers to avoid duplication.

The exclusion criteria were as follows:

‐ Literature with no available full‐texts including the conference papers and abstracts;
‐ Literature with the main focus of nonhuman experiments of any kind like in vitro studies, animal trials, or literature without justifying details;
‐ Reviews, systematic reviews, or meta‐analyses;
‐ Case reports.

### 2.4 Data extraction

Two independent investigators summarized and extracted the following information from the included publications: The first author's ID (Reference), year, and type of publication (e.g., cross‐sectional study), country of study, the sample size of the study, patient mean age and gender, sampling site, measured viral load, and disease outcome; the data were further gathered in a specifically designed sheet and organized into tables.

### 2.5 Quality/risk of bias assessment

We used the Newcastle–Ottawa Scale to assess the quality of the studies.^25^ This scale yields a total score out of 9 to the studies based on their selection, comparability, and exposure/outcomes.

## 3 RESULTS

**Figure 1:**
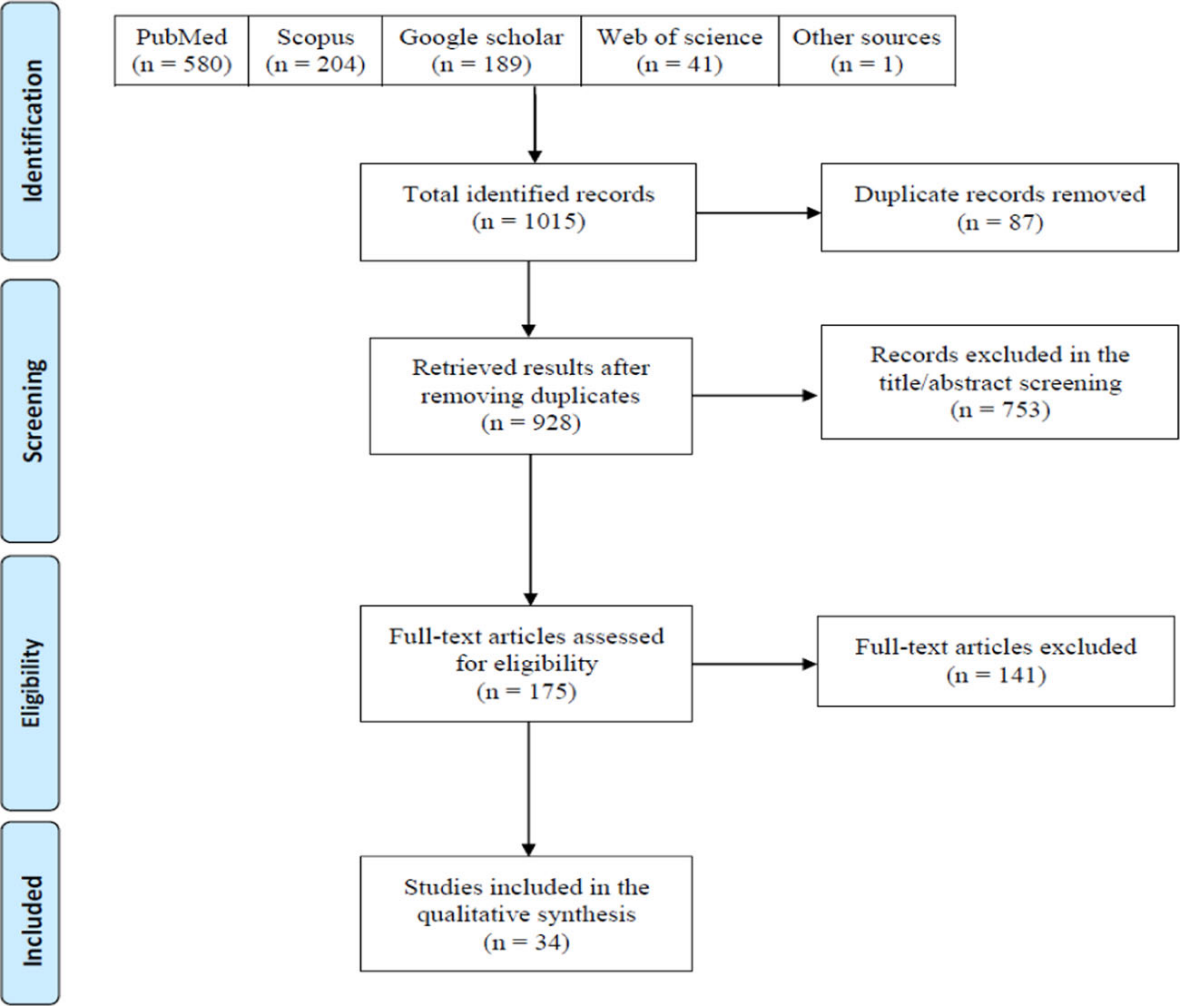
Prisma flow diagram of the study's selection process

The search strategies resulted in 1015 records, being 928 remaining after removing the duplicates. Of which, 753 records were excluded in the title/abstract screening, and 175 full texts were reviewed. Finally, 34 studies met the eligibility criteria to be included after full‐text screening (Figure 1). Most of the studies were from China (*n* = 7); three studies per following countries: Japan, Spain, Turkey, and the USA; two studies per following countries: Italy, South Korea, and Switzerland; and one study for the following countries: Brazil, Czech Republic, England, France, Germany, India, Israel, and Singapore (Table 1). The studies had overall acceptable quality, all of them scoring 4 and above (Table 2).

**Table 1:** Summary of the findings of the included studies

ID	First author (reference)	Type of study	Publication year	country	Study population	age	Gender	Sampling method	Viral load and its association with disease severity	Sign/symptom	Comorbidities	Lab test	Clinical outcome	Transmission	Important finding
1	Aoki et al.^26^	Cross‐sectional	2021	Japan	24	N/A	Female/male	RT‐PCR	There was a high correlation between viral load calculated using the RT‐PCR cycle threshold value and antigen concentration. The tendency to decrease antigen concentration over time after disease onset is associated with viral load. *C*_t_ value: 25	N/A	N/A	N/A	N/A	N/A	SQT is fully compatible with RT‐PCR and should be useful in diagnosing COVID‐19 in any clinical setting
								Nasopharyngeal swab							
2	Aydin et al.^27^	Case series	2021	Turkey	125	62,1	48.8% male	RT‐PCR	The effect of SARS‐CoV‐2 viral load on saliva and other substances was not found in their prognosis. *C*_t_ value: 22.28	N/A	Hypertension, COPD, DM, malignancy, immune deficiency, cardiovascular disease, and asthma	N/A	N/A	N/A	The viral load of saliva in the early stages of COVID‐19 infection may have a high prognostic value in predicting disease progression in patients over 45 years of age. Saliva is a good substance in COVID‐19 screening
								Oronasopharyngeal (ONP) samples and saliva samples							
3	Berastegui‐Cabrera et al.^28^	Cross‐sectional	2021	Spain	72	66	56% male	RT‐PCR	No association was found between viral load in NP samples and the presence of SARS‐CoV‐2 RNAemia. The absence of differences in NP viral load between patients with SARS‐CoV‐2 RNAemia and without it proves that the clinical development index of COVID‐19 patients is better than that of NP viral load. The median viral load in NP swabs = 6.98 log_10_ copies/ml (IQR, 5.15–8.20)	Arthromyalgias, coryza, cough, dyspnea, headache, odynophagia, diarrhea, anosmia, weakness, and dysgeusia	Chronic kidney disease, solid‐organ transplantation, connective tissue disease, and chronic liver disease	Leukocytes: 5.22, 7.00, Neutrophils: 3.49, 4.79, Lymphocytes: 0.58, 1.36, Platelets: 158, 248, Hemoglobin: 13, 13.8, AST: 37, 26, ALT: 33, 23, Bilirubin: 0.59, 0.46, Sodium: 2, 4, Potassium: 2,1, Creatinine: 4, 6, C‐reactive protein: 97.9, 44.9, Ferritin: 625.6, 442, D‐dimers: 1430, 620, LDH: 450, 251.5,	ARDS, multiple organ failure, IMV, ICU admission, mortality	N/A	The presence of RNAemia SARS‐CoV‐2, in the first emergency assessment, is more common in patients with severe chronic underlying disease, such as chronic liver disease and solid organ transplantation, with viral load in the upper respiratory tract and with adverse outcomes
								Nasopharyngeal swabs							
4	Buetti et al.^29^	Cross‐sectional	2021	Switzerland	90	62.5	78.9% male	RT‐PCR Nasopharyngeal swab	Viral load (log_10_ copies/ml), median [IQR]: 3.3 [1.8; 5.2] That viral load in the LRT was associated with the 6‐week mortality		Cardiovascular, Immunosuppression, DM, Renal failure, Cancer, and Chronic respiratory failure	N/A	N/A	N/A	Delay in LRT virus averaged approximately 30 days in critically ill patients, and viral load in LRT was associated with 6‐week mortality
5	Buder et al.^30^	Cohort	2021	Germany	59	Median: 48 years	49%	Quantitative real‐time PCR of respiratory samples	Median viral load (IQR): 6.80 × 10^4^ (4.75 × 10^3^–1.81 × 10^6^) RNA copies/ml	N/A	10 patients had immunosuppression	N/A	34 outpatient, 20 admitted to ICU	Higher viral load correlated with a higher chance of viral transmission	SARS‐CoV‐2 infectivity correlated with viral load, with the best predictor of infectivity being viral loads above 1.0 × 10^7^ RNA copies/ml. The probability of virus isolation from respiratory samples also correlated positively with viral load. Seroconversion terminated SARS‐CoV‐2 infectivity
6	Cho et al.^31^	Pospective observational	2020	China	75	36.4 ± 16.3	48% male	RT‐PCR Nasopharyngeal and deep throat swabs	There was no correlation between the recovery time of olfactory or gustatory disorders and the Ct value of PCR was sampled indirectly from nasopharyngeal swabs and deep throat reflected the viral load of SARS‐CoV‐2. *C*_t_ value: 28.3 ± 6.7	Rhinorrhea, Purulent nasal discharge, Taste change, Nasal blockage, Epistaxis, Cough, Fever, Dyspnea, and smell change.	N/A	N/A	N/A	N/A	There is no association between severity and improvement of olfactory and taste disorders with SARS‐CoV‐2 viral load
7	Chua et al.^32^	Cross‐sectional	2021	China	91	Asymptomatic Male:8.6 (4.3–11.0), Symptomatic Mean (IQR): 9.2 (4.0–15.0)	Asymptomatic 57.1% male, Symptomatic 44.4% male	RT‐PCR Nasopharyngeal swab (NPS), and saliva samples collected on admission	The onset days of symptoms for all patients were inversely related to the NPS and saliva viral loads. Viral load (log_10_ copies/ml): lymphopenia (NPS, Saliva): 6.7, 5.8 viral load (log_10_ copies/ml):: nonlymphopenia (NPS, Saliva): 6.2, 4.9		N/A	Total white cell count (×10^9^/L): 6, 5.8‐Hemoglobin (g/dl): 12.8, 13.2‐Platelets (×10^9^/L): 258.4, 278.1‐Urea (mmol/L): 3.4, 3.9‐Creatinine (µmol/L): 41.6, 44.9‐Creatine Kinase (U/L):122.5, 99.7‐Troponin I (ng/l): 1.9, 11.3‐C Reactive Protein (mg/dl): 1.4, 1.7‐Erythrocyte Sedimentation Rate (mm/h):8.6, 12‐	N/A	N/A	Salivary viral loads in hospitalized children with clinical and immune profiles are better than NPS
8	de la Calle et al.^33^	Cross‐sectional	2021	Spain	455	64.9 ± 18.1	56% male	rRT‐PCR nasopharyngeal	Patients with respiratory failure had a higher viral load at admission than those who did not. Low viral load (*C*_t_ > 30), Intermediate viral load (*C*_t_ 25–30): 1.81, high viral load (*C*_t_ < 25): 2.99	Fever, Vomiting, Cough, Tachypnea, Diarrhea, SpO2 < 90% air room, Myalgia and Dyspnea	Cardiovascular disease, chronic renal disease, chronic lung disease, DM, immunosuppression, obesity, current or former smoker, and chronic liver disease	LDH (U/L): 326.6, GOT (U/L): 32, GPT (U/L): 25, CPK (U/L): 86, TnT (U/L): 10.5, C‐reactive protein (mg/dl): 7.7, Ferritin (mg/dl): 699, D‐dimers (ng/ml): 664	Need for supplemental oxygen, ARDS, noninvasive mechanical ventilation, ICU admission, Septic shock, Prone position, MACE event, Acute kidney injury (AKI), Venous thrombosis, Hepatitis, Respiratory failure, Invasive mechanical ventilation, and mortality	N/A	The SARS‐CoV‐2 viral load, measured by Ct value of rRT‐PCR in pharyngeal swabs at admission, is a good indicator of the prognosis for respiratory failure
9	He et al.^34^	Cohort	2020	China	23	41	43.5% males	qRT‐PCR Pharyngeal swab	Minimum viral load: 40 *C*_t_. Asymptomatic type patients had lower viral loads than common and severe types	Fever, cough, nasal congestion, dizziness, fatigue, arthralgia,	human endogenous retrovirus‐H (Hervs) and Human picobirnavirus (HPBV)	Patients with severe disease had more abnormal laboratory test results (including leukopenia and lymphocytopenia)	no significant correlation was observed between age and Ct value also no association between Ct value and severity of illness was observed. Significant positive relation has been detected between peak viral load and severity of illness.	Weaker transmission capacity of asymptomatic cases due to the lower viral load	Asymptomatic type patients had lower viral loads than common and severe types
10	Jacot et al.^35^	Cross‐sectional	2020	Switzerland	N/A	0‐99 years	Female/male	RT‐PCR Nasopharyngeal swab	Range: 3–10 log copies/ml. Median: 6.78 log_10_ copies/ml In the first period of covid‐19 outbreak viral load was higher SARS‐CoV‐2 viral load seem to be a substandard predictor of disease outcome, COVID‐19 disease severity is not significantly related to viral replication in the upper and lower respiratory tracts	Fever cough	N/A	N/A	In the first period of covid‐19 outbreak viral load was higher	below 1000 copies/ml values can be considered at slight risk of transmission	Despite there are significant differences between viral loads of different viruses, SARS‐Cov‐2 had a alike viral load to Respiratory syncytial virus and influenza B than other coronaviruses
11	Jain et al.^36^	Comparative	2021	India	200	group A 35.23 ± 11.99, group B 35.32 ± 12.92	60% male	RT‐PCR Nasopharyngeal swab	Group A with olfactory and taste dysfunction: 24.43 *C*_t_. Group B without OTD: 27.39 *C*_t_. The patients with taste and olfactoryimpairment at diagnosis had more viral load than patients without taste and olfactoryimpairment	Loss of smell and taste malaise sore throat cough fever nasal discharge	N/A	RT‐PCR was utilized to test The COVID‐19, with 3 gene detection: RdRp (RNA‐dependent RNA polymerase), E (Envelope encoding) gene, and N (Nucleocapsid encoding) gene. For analysis cycle threshold was utilized.	N/A	N/A	The patients with OTD at diagnosis had more viral load than patients without OTD
12	Kam et al.^37^	Cohort	2021	Singapore	17	7.7	Female/male	RT‐PCR Nasopharyngeal swab	Symptomatic: 28.6 *C*_t_. Asymptomatic: 36.7 *C*_t_ higher viral loads was seen in symptomatic children in comparison to asymptomatic children	Upper respiratory tract symptoms with mild sickness signs	N/A	N/A	patients with mild and severe chest CT involvement had significantly lower viral load in comparison to patients with no chest CT lesions.	symptomatic children in had high viral load in the first stage of sickness indicates the transmission potential of presymptomatic children.	Children with symptomshad higher viral loads than children without symptoms
13	Karahasan Yagci et al.^38^	Cohort	2020	turkey	730	35	49.9% female	RT‐qPCR Nasopharyngeal swab	Without CT scan involvement: 24.9 mild CT involvement: 27.8 moderate CT involvement: 29.4 severe CT involvement: 27.9. The oppositecorrelation of chest CT Total severity score (TSS) and viral load was seen. Significantly higher viral loads was observed in patients with no chest CT lesions in comparison to patients with mild and severe chest CT involvement	Fever, cough and dyspnea	Hypertension, diabetes mellitus, cardiovascular disease, chronic obstructive pulmonary diseases (COPD), cancers, HIV, collagenosis, and chronic liver disease	N/A	284 (39%) patients were admitted to hospital and 27 of patientsexpired during the hospitalization.	N/A	The oppositecorrelation of chest CT total severity score (TSS) and viral load
14	Kawasuji et al.^39^	Case‐control	2020	Japan	28	Median age: 45.5 years	53.6% male	rRT‐PCR Nasopharyngeal swab	33.6 ± 5.5 *C*_t_. A significant viral load and recovery time differencewas observed between patients with pulmonary involvement and patients without pulmonary involvement	N/A	N/A	N/A	Significantly higher viral load at the beginning of sampling in symptomatic patients than in asymptomatic patients was observed. Also, Children had significantly higher viral load than adults in the beginning of sampling.	A high nasopharyngeal viral load can be connected to the secondary transmission of COVID‐19	Secondary transmission of COVID‐19 can be related to high nasopharyngeal viral load. Additionally, the viral load can help describe why transmission is observed in some patients, but not in others, particularly among patients who live in same house
15	Kim et al.^40^	Retrospective	2021	South Korea	106	Mean age: 28.0 ± 9.3 years	43.4% male	RT‐PCR Nasopharyngeal/oropharyngeal swab	33.6 ± 5.5 *C*_t_. Viral load and recovery time were significantly different between pulmonary involvement patients and patients without pulmonary involvement was observed	Cough, fever, headache, hyposmia, rhinorrhea, sputum, muscle pain, diarrhea, chest pain, ocular pain	Rhinitis, asthma, migraine, iron deficiency, anemia, hyperlipidemia, endometriosis, depression disorder, hair loss, atopic dermatitis	N/A	Recovery times were significantly slower in the patients with pulmonary involvement than patients without involvement.	N/A	Viral load and recovery time were significantly different between pulmonary involvement patients and patients without pulmonary involvement was observed. The cycle threshold cutoff value for the existence of pneumonia was 31.38
16	Kociolek et al.^41^	Retrospective	2020	USA	817	0‐17 years	52.1% male	RT‐PCR Nasopharyngeal swab	Asymptomatic children: 2.0 × 10^3^ copies/ml symptomatic children: 1.3 × 10^7^ copies/ml. In children without symptoms lower viral load was found in their nasopharynx/oropharynx than children with symptoms	Cough, fever/chills, dyspnea, pharyngitis, loss of taste or smell, headache, abdominal pain, diarrhea, fatigue, myalgias, congestion/rhinorrhea, nausea/vomiting, rash, or conjunctivitis	Immunocompromised = 51. Diabetes = 19	N/A	Ct values were significantly higher in children without symptoms than children with symptoms. Also, significantly lower viral loads was observed in asymptomatic than symptomatic children.	N/A	Asymptomatic children had low viral loads in their nasopharynx/oropharynx than children with symptoms
17	Kriegova et al.^42^	Prospective	2021	Czech Republic	1038	50.0 ± 3.3	Female/male	RT‐PCR Nasopharyngeal swab	Asymptomatic and mild group 23.65 (±7.62) *C*_t_. Moderate group 27.68 (±6.98) *C*_t_. Severe and critical group 26.52 (±4.82) *C*_t_. High levels of virus in the respiratory tract and excessive producing of chemokines and cytokines between first 2 weeks from the onset of symptoms were significantly related to severity of the COVID‐19	N/A	N/A	N/A	self‐conductnasal‐swab in combination with direct RT‐qPCRare easy, low‐cost and quick CoV‐2 testing method which could significantly increase the extent of the teststrategies which are needed to control the expansion of COV‐19 during and post‐pandemic era	N/A	High levels of virus in the respiratory tract and too much productionof chemokines and cytokines and between the first two weeks from the onset of symptoms were significantly related to severity of the COVID‐19
18	Kwon et al.^43^	Prospective	2020	South Korea	31	32‐72 years	58% female	Nasopharyngeal swab RT‐PCR	Initial viral load at five toten days from onset of symptoms in the asymptomatic and mild group, moderate group, and the severe and critical group was 32.65 (±7.62), 27.68 (±6.98), and 26.52 (±4.82) cycles	Fever, chill, cough, sputum, sore throat, dyspnea, rhinorrhea chest pain, headache, myalgia, nasal congestion, hyposmia, hypogeusia, pneumonia	Diabetes mellitus, hypertension, chronic lung disease, chronic liver disease, obesity (body mass index > 25), smoking	Old age, initial low WBC count, low platelet count, high CRP level, and fever were identified as factors associated with severity	Early increases in type I IFN response might be involved in the pathophysiology of severe COVID‐19 by eliciting subsequent excessive responses of multiple cytokines and chemokines	N/A	Higher viral load, stronger antibody response, and excessive inflammation at first two weeks from onset of symptoms are related to the COVID‐19 severity
19	Le Borgne et al.^44^	Retrospective	2021	France	287	50.0 to 73.0, median age: 63.1	65.8% male	Pharyngeal swabs qRT‐PCR	4.76 (3.29–6.06) log_10_ copies/reaction Nasopharyngeal viral load measured by RT‐PCR during beginning emergency department (ED) viral load is not predictor of severity and mortality in COVID‐19 patients	N/A	Hypertension, cardiovascular disease, diabetes mellitus, renal insufficiency, dialysis, COPD, malignancies, immunotherapy, corticosteroids	At emergency department admission, patients who didn't survive in comparison to survived patients. had significantly higher C‐reactive protein (122 vs. 74 mg/L, *p* = .007) and creatinine (*p* = .036). Nonsurvivors were also more likely to present with anemia (*p* = .003) and lymphopenia (*p* = .02) than survivors	Forty‐two patients (14.6%) died.		Nasopharyngeal viral load was measured by RT‐PCR at emergency department admission viral load isn't predictor of severity and mortality in COVID‐19 patients
20	Piubelli et al.^45^	Cross‐sectional	2021	Italy	273	N/A	Female/male	RT‐PCR Nasal and Pharyngeal swabs	Viral load decreased during 2 months of quarantine (*C*_t_ decreased from 24 to 34). Alongside, the number of patients who need intensive care significantly decreased because of the reduction of viral load	N/A	N/A	More probable in high‐transmission setting compared with low‐transmission setting	ICU admission (5.3%)	N/A	N/A
21	Rauch et al.^46^	Cohort	2021	USA	1808	27.3 ± 11	53% male	RT‐qPCR and CRISPR‐based assay Nasopharyngeal swab	Viral load = 286–510,000 copies/μl. The shift of viral load is shown in those who stayed at home	Nasal congestion, sore throat, fatigue, anosmia	N/A	8 positive participants by CRISPR‐based assay and 9 by RT‐qPCR were detected	All were alive at the end of the study	N/A	The prevalence of SARS‐CoV‐2 in cohort 2 was changed and it was because of decreased community restrictions and increased social interactions
22	Sarkar et al.^47^	Cross‐sectional	2020	India	138	N/A	Female/male	RT‐PCR Nasopharynx swab (NPS) and oropharynx swab (OPS)	In those with *C*_t_ values between 17 and 23, patients had severe infections	N/A	N/A	N/A	N/A	In high viral load cases, the rate of transmission was 8‐times more than low viral load cases. Patients with Ct above 33‐34 were not contagious	In individuals with high viral load, the possibility of transmission was almost 8 times higher compared to low viral load individuals. Of those who were infected, 7% had a high viral load, 9% moderate viral load, and 84% low viral load based on Ct values. The probability of transmission in those with high viral load was 6.25 in comparison with law viral load with 0.8
23	Shlomai et al.^48^	Cross‐sectional descriptive	2020	Israel	170	62	58% Male	Nasopharyngeal samples RT‐PCR	Viral load was significantly higherin ventilated and nonsurvivors patients (eightfold more than other patients). Low viral load was associated with decreased risk of mortality and intensive care	Hypoxemia	N/A	N/A	21 death	N/A	Viral load was directly linked to hypoxemia. Viral load was significantly related toblood oxygen saturation. The patient's age significantly correlated with viral load
24	Shrestha et al.^49^	Cohort	2020	USA	230 health care personnel (HCP)	N/A	Male 36%	PCR Nasopharyngeal swab	Viral load in 2 or 3 days after onset of symptoms was the peak. Time since onset of symptoms was significantly related to viral load	N/A	Chronic lung disease, current smoker, chronic heart disease, hypertension, liver cirrhosis, immunocompromised, diabetes mellitus, chronic kidney disease	N/A	N/A	N/A	86.5% of transmission potential was in the first 5 days since onset of symptoms
25	Singanayagam et al.^50^	Cross‐sectional	2020	England	754 samples from 425 symptomatic cases	0‐100 years old	Female/male	RT‐PCR Nose, throat, combined nose‐and‐throat and nasopharyngeal swabs	There was no difference in *C*_t_ value between asymptomatic (*C*_t_ = 31.23), mild to moderate (*C*_t_ = 30.94), and severe cases (*C*_t_ = 32.55). In the first week of onset of symptoms, viral load was higher than the second week	N/A	N/A	In 42% of cases, culture was positive. The culture positivity during the first week of infection was significantly higher than the second week	N/A	N/A	Cases in the 81–100 year age group were more asymptomatic than other groups
26	Soria et al.^51^	Cohort	2020	Spain	448	71.04 ± 18.29	45.7% male	RT‐PCR Nasopharyngeal swabs	Mean *C*_t_: mild (35.75 ± 0.45), moderate (32.69 ± 0.37), severe (29.58 ± 0.70). Viral load is a predictor of disease severity. High virus loading worsens the prognosis of the disease. *C*_t_ value was significantly law in the severe group in comparison with the moderate and mild group	N/A	Hypertension, cardiovascular disease, diabetes. Obesity, asthma, COPD	N/A	Cases of the severe group include 23% of total cases and all of them were admitted. Also, 18.3% died during 90 days after diagnosis, 75 cases in the severe group, three cases in moderate, and four in the mild group	N/A	N/A
27	To et al.^52^	Cohort	2020	China	23	62	56.5% male	RT‐qPCR Oropharyngeal saliva samples	The median viral load was 5 × 2 log_10_ copies/ml. The first week after the onset of symptoms, the viral load is high but decreases over time	Fever (96%), cough (22%), chills (17%), dyspnea (17%), runny and blocked nose, sore throat, chest discomfort, nausea, diarrhea, myalgia, malaise. In 15 (65%) CXR abnormalities were seen. In 17 (74%) multifocal ground‐glass lung opacities were seen	48% had clinical medical illnesses including hypertension and diabetes	Those patients who had comorbidities had a lower anti‐RBD IgG OD compared to those without comorbidities	Five patients were admitted to ICU, two of them required intubation, and also two of them died	N/A	Older age was associated with a higher viral load. The antibody response occurred 10 days or later since the onset of symptoms
28	To et al.^53^	Cross‐sectional	2020	China	12	62.5	58% male	RT‐qPCR Nasopharyngeal or sputum specimen	The median viral load was 3.3 × 10^6^ copies/ml. On the first day of hospitalization viral load was slightly higher than other days. After day 11 viral load started to shed till being undetectable	N/A	N/A	According to viral culture, saliva contains live viruses and potentially can transmit the virus	At the end of the survey, all patients were alive	N/A	Saliva can be obtained from the patient without invasive procedure and it leads to reduce in nosocomial transmission of the virus
29	Trunfio et al.^54^	Retrospective cross‐sectional	2021	Italy	200	56	58% male	RT‐PCR Nasopharyngeal swab	Viral load was associated with the severity of the disease	Gastrointestinal, neurological, respiratory, and systemic involvement, headache, olfactory and gustatory dysfunction, nausea and vomiting, diarrhea, fever, arthralgia, asthenia and malaise, cough, dyspnea, pharyngitis, and runny nose	Participants of group A (*C*_t_ ≤ 20) had at least one comorbidity that was significantly different from the other two groups. Hypertension, COPD, asthma, obesity, active smoking, diabetes, cancer	N/A	36.5% of cases were isolated at home and 63.5% were admitted to the hospital. Of those admitted, 16% died (including 20 cases in group A, 7 cases in group B, 5 cases in group c). 5% of all cases required intubation	N/A	Group A (*C*_t_ ≤ 20) washospitalized more than group C (*C*_t_ > 28). COVID‐19 severity and worse outcomes were significantly higher in group A compared with the other two groups (B: 20 < *C*_t_ < 28). There was no association between viral load and prevalence of olfactory/taste disorder
30	Tsukagoshi et al.^55^	Cross‐sectional	2021	Japan	286	39 ± 35	56.3% male	RT‐qPCR Nasopharyngeal swab	In fatal cases 3.57 × 10^9^ ± 4.70 × 10^9^ copies/ml; in survived cases 3.92 × 10^8^ ± 1.60 × 10^9^ copies/ml; in asymptomatic 4.92 × 10^7^ ± 1.48 × 10^7^ copies/ml. In fatal cases, viral load was significantly higher than symptomatic and asymptomatic cases. Poor prognosis in elderly patients was predicted in those with a high viral load	Fever, sore throat, cough	N/A	N/A	5.2% of cases died	N/A	Pneumonia was more common in patients who died than in those who survived
31	Wang et al.^56^	Cross‐sectional	2020	China	23	56	82.6% male	RT‐PCR Nasal swab, pharyngeal swab, sputum	In severe cases in comparison with mild cases, the viral load peak was significantly higher	N/A	N/A	N/A	43.5% of cases admitted to ICU	N/A	N/A
32	Faíco‐Filho et al.^57^	Cohort	2020	Brazil	875	48	49.1% male	RT‐PCR Nasal swab	Samples with *C*_t_ values <40 were considered positive. Survivors presented a significantly higher initial *C*_t_ value than that of nonsurvivors Mortality rates were 46% among patients with a high viral load (*C*_t_ < 25) and 22% among patients with a low viral load		N/A	N/A	The higher the viral load, the worse the disease and the poorer the consequences	N/A	the Ct value could be used as a tool to help with the identification of patients at a higher risk for severe consequences
33	Guo et al.^58^	Cohort	2020	china	195	49.24 ± 15.99	48.2% males	RT‐PCR Nasopharyngeal swab	More severe patients seem to have a higher initial viral load. a significant increasing trend of initial viral load versus illness severity	Higher maximum body temperature within 24 h after hospitalization anddurationoffever (days) correlation with severe disease	Hypertension, Diabetes mellitus, Cardiovascular disease, Cerebrovascular disease, Chronic kidney disease	Higher plasma C‐reactive protein (CRP), D‐dimer, procalcitonin (PCT), and aspartate aminotransferase (AST); larger count of white blood cells (WBC) and neutrophil (NE), but relatively reduced lymphocyte count. A higher NE to lymphocyte ratio (NLR) was seen at a severe disease	N/A	N/A	Age, fever, peak body the temperature in 24 h after hospitalization, CRP, WBC, NE, NLR, AST, D‐Dimer, and PCT are positively correlated with severity, Patients with higher upper respiratory tract viral load at admission are more likely to develop severe symptoms and may need more aggressive treatment
34	Hasanoglu et al.^59^	Retrospective study	2020	Turkey	60	33.9	48% males	RT‐PCR Saliva, urine, blood, and anal swab samples	The viral load of standards synthetic SARS‐CoV‐2 RdRp fragment/ml was between 2.5 × 10^2–5^ copy/ml. No significant difference in the probability of PCR positivity across symptomatic and asymptomatic patients was found. PCR positivity does not always indicate infectivity	Cough and fatigue were the most observed symptoms on admission, 51.7%, and 30.5%, respectively	At least one comorbidity was present in 8 (13.3%) patients	N/A	Factors associated with poor prognosis are found to be significantly correlated with low viral load	N/A	A significant decrease in viral load of nasopharyngeal/oropharyngeal samples was observed with increasing disease severity

Abbreviations: LRT, lower respiratory tract; NPS: nasopharyngeal swab.

**Table 2:** Quality assessment for the included studies using the Newcastle–Ottawa Scale

**The first author (reference)**	**Selection (out of 4)**	**Comparability (out of 2)**	**Exposure/outcome (out of 3)**	**Total score (out of 9)**
Aoki et al.^26^	***	–	**	5
Aydin et al.^27^	***	–	**	5
Berastegui‐Cabrera et al.^28^	****	**	*	7
Buetti et al.^29^	***	**	***	8
Buder et al.^30^	***	**	**	7
Cho et al.^31^	**	–	**	4
Chua et al.^32^	**	–	***	5
de la Calle et al.^33^	***	**	***	8
He et al.^34^	**	**	**	6
Jacot et al.^35^	***	–	**	5
Jain et al.^36^	****	**	**	8
Kam et al.^37^	***	–	**	5
Karahasan Yagci et al.^38^	***	**	***	8
Kawasuji et al.^39^	**	–	**	4
Kim et al.^40^	***	–	**	5
Kociolek et al.^41^	****	**	**	8
Kriegova et al.^42^	****	–	**	6
Kwon et al.^43^	***	**	***	8
Le Borgne et al.^44^	****	**	**	8
Piubelli et al.^45^	***	**	**	7
Rauch et al.^46^	****	**	***	9
Sarkar et al.^47^	**	**	**	6
Shlomai et al.^48^	**	–	**	4
Shrestha et al.^49^	**	**	**	6
Singanayagam et al.^50^	****	**	***	9
Soria et al.^51^	***	–	***	6
To et al.^52^	***	**	**	7
To et al.^53^	***	**	**	7
Trunfio et al.^54^	***	**	***	8
Tsukagoshi et al.^55^	**	–	**	
Wang et al.^56^	**	–	**	4
Faíco‐Filho et al.^57^	****	**	***	9
Guo et al.^58^	***	**	***	8
Hasanoglu et al.^59^	**	**	**	6

Most of the studies included adults and had a similar share of men and women. The vast majority of the studies have utilized real‐time RT‐PCR of the nasopharyngeal/respiratory swabs to report viral load. Viral load was usually reported in two categories; cycle threshold (*C*_t_) and log_10_ RNA copies/ml. Studies have reported viral load in several groups: mild, moderate, and severe patients, symptomatic versus asymptomatic patients, and groups sorted by age. The results were inconsistent; while some studies found a significant relationship between SARS‐CoV‐2 viral load and severity of illness, other studies were against it (Table 1).

## 4 DISCUSSION

SARS‐CoV‐2, the new coronavirus accountable for COVID‐19, was first detected in China in late 2019 and then spread out globally. The WHO declared this disease a public health emergency of international concern on January 30, 2020. Although having the potential of causing severe pneumonia, SARS‐CoV‐2 can also involve various organs and cause physical symptoms such as fever, cough, and dyspnea, as well as psychological and gastrointestinal symptoms. Several interventions and measures have been implemented to restrict the spread of the virus and control the situation, such as community education, border controls, lockdown, social distancing, wearing masks in public, hand hygiene, and schools shut down. These public health efforts not only slowed down SARS‐CoV‐2 transmission but also caused a decrease of mortality rate.^1^, ^12^

In the present study, the main hypothesis along with two minor ones was discussed against the similar available studies. The main hypothesis recommended a potential relationship between the viral load and the severity of the disease. The minor hypotheses, which were also frequently reported in the included studies, are the relation between the age and the viral load as well as the relation between viral load and virus transmissibility. Symptoms included in the table were aimed to represent the severity of the diseases and the included comorbidities were to avoid the bias of imposture relation between severity and the viral load.

For both hypotheses, the key method of measuring the viral loads was the RT‐PCR. Viral nucleic acid detection by RT‐PCR assays is the gold standard for the diagnosis of COVID‐19. Using this technique, we can gain an indirect viral load value (*C*_t_) easily and immediately after diagnosis.^33^ The main hypothesis could be explained by the association between viral load and inflammatory factors that are also clearly connected with the disease severity. It is well‐known that excessive release of proinflammatory cytokines and chemokines contributes to the severity of clinical outcomes in various infections. Therefore, our findings that the plasma concentrations of IFN‐α, IFN‐γ, IP‐10, MIG, and IL‐6 were elevated in the severe and critical cases at 5–10 days from symptom onset suggest that the higher plasma concentrations of proinflammatory cytokines after approximately a week from symptom beginning may have a role in the enhancement of severity. Intriguingly, a recent longitudinal study showed that plasma IFN‐α continued to be high in patients with severe COVID‐19, whereas it dropped in those with moderate COVID‐19 during their clinical course.^43^

Similar to our findings, He et al.,^34^ have identified that higher viral load was positively associated with COVID‐19 severity. This finding highlights the importance of monitoring the viral kinetics to identify patients at greater risk of progressing to severe pneumonia. Similarly, Guo et al.,^58^ have found that the upper respiratory tract viral RNA load of SARS‐CoV‐2 at the time of hospital admission is an independent predictive factor of COVID‐19. However, there were some studies with inconsistent results. The study performed by Hasanoglu et al.,^59^ is an example of this controversy. They demonstrated that asymptomatic patients have higher SARS‐CoV‐2 viral loads than symptomatic patients and unlike in the few study in the literature, a major decrease in viral load of nasopharyngeal/oropharyngeal samples was observed with increasing disease severity. Similarly, Cho et al.^31^ have found that both severity and recovery from these symptoms have no associations with the viral load of SARS‐CoV‐2. Le Borgne et al.,^44^ have also found that respiratory viral load measurement on the first nasopharyngeal swab (by RT‐PCR) during initial ED management is neither a predictor of severity nor a predictor of mortality in SARS‐CoV‐2 infection.

To support our minor hypotheses suggesting the association between viral load and patient's age, the findings from the study by To et al.,^52^ suggested no relationships between severity of disease and viral load; their study only showed that the median viral load was 1 log_10_ higher in severe cases than in mild cases, but on the other hand, they found a direct connection between age and viral load. Similarly, Shlomai et al.^48^ have found that low viral load was independently associated with reduced risk for mechanical ventilation and mortality; and interestingly, patients' age also correlated positively with the viral load. Aydin et al.^27^ found that viral load detected in saliva in the early symptomatic period of infection may have a prognostic value in showing the course of the disease in patients over 45‐year‐old. Overall, the studies found a positive correlation between patients' age and viral load. This finding might be a rationale for any possible relationship between viral load and increased disease severity, as older age is related to worse COVID‐19 outcomes.^11^ It also raises the alarm that older patients may be more likely to transmit the virus.

In the present study, the final hypothesis suggesting the association between viral load and the COVID‐19 infectivity could be supported by the findings of Kawasuji et al.'s study, which suggested that a high nasopharyngeal viral load may contribute to the secondary transmission of COVID‐19.^39^ Similarly, Sarkar et al. found that 84% of cases had low viral load and practically will spread the virus even to very few their contacts, demonstrating the connection between viral load and transmission.^47^ Buder et al. have also reported similar results that merely having no symptoms is not enough for recognizing whether the patients have the ability of transmission or not. They found that SARS‐CoV‐2 positively correlated with the infectivity of the patients, regardless of whether they are symptomatic or not.^30^ Therefore, viral load is probably one of the factors influencing SARS‐CoV‐2 transmission.

There are some limitations in the present study. First and most important, a meta‐analysis was not conducted due to the significant heterogeneity that existed between the included studies. Furthermore, there were few studies on some of the discussed matters and this may decrease the validity and reliability of reported outcomes. However, this study may provide relevant insights for future research to conduct original studies and/or meta‐analyses to precisely determine the relationship between viral load and disease severity, and, in addition, to explore further discussed topics in this review, such as the correlation between age and SARS‐CoV‐2 viral load.

## 5 CONCLUSION

We have discussed three different hypotheses related to the viral load of COVID‐19. The results were inconclusive about the relationship between COVID‐19 severity and viral load, as a similar number of studies either approved or opposed this hypothesis. However, the included studies found a positive association between age and viral load. The higher viral load also appeared to be associated with the higher transmissibility of the disease. Nevertheless, such findings require careful meta‐analyses to be confirmed.

## CONFLICT OF INTERESTS

The authors declare that there are no conflict of interests.

## AUTHOR CONTRIBUTIONS

*Conception and design of the study*: Esmaeil Mehraeen, SeyedAhmad SeyedAlinaghi. *Acquisition of data*: Amirali Karimi, Nazanin Janfaza, Soheil Dehghani, and Fatemeh Afroughi. *Analysis and interpretation of data*: Pegah Mirzapour and Alireza Barzegary. *Drafting the article*: Amir Masoud Afsahi, Zahra Pashaei, Hengameh Mojdeganlou, Amirali Karimi, Pedram Habibi, Alireza Barzegary, Amirata Fakhfouri, Pegah Mirzapour, Nazanin Janfaza, Soheil Dehghani, Fatemeh Afroughi, Mohsen Dashti, Sepideh Khodaei, and Omid Dadras. *Revising it critically for important intellectual content*: SeyedAhmad SeyedAlinaghi, Esmaeil Mehraeen, and Omid Dadras. *Final approval of the version to be submitted*: Esmaeil Mehraeen, Omid Dadras, SeyedAhmad SeyedAlinaghi, Fabricio Voltarelli, and Jean‐Marc Sabatier.

## Data Availability

The authors stated that all information provided in this article could be share.
